# Study To Evaluate the Performance of a Point-of-Care Whole-Blood HIV Viral Load Test (SAMBA II HIV-1 Semi-Q Whole Blood)

**DOI:** 10.1128/JCM.02555-20

**Published:** 2021-02-18

**Authors:** Gary Brook, Tetiana Stepchenkova, Innocent M. Ali, Sandra Chipuka, Neha Goel, Helen Lee

**Affiliations:** a Department of HIV and Sexual Health, Central Middlesex Hospital, London North West University Healthcare NHS Trust, London, United Kingdom; b Department of HIV, Kiev City AIDS Center, Kiev, Ukraine; c Department of Biochemistry, University of Dschang, West Region, Cameroon; d National Microbiology Reference Laboratory, Harare Central Hospital, Harare, Zimbabwe; e Diagnostics for the Real World, Cambridge, United Kingdom; f Department of Haematology, University of Cambridge, Cambridge, United Kingdom; Rhode Island Hospital

**Keywords:** HIV, antiretroviral therapy, diagnostics, point-of-care

## Abstract

Remote areas of many low and middle income (LMI) countries have poor access to HIV viral load (HIV VL) testing. The SAMBA II (simple amplification-based assay) Semi-Q whole-blood test (Diagnostics for the Real World [DRW], Cambridge, UK) is a point-of-care assay, which uses leucodepletion technology to allow whole-blood testing in remote settings. A total of 1,540 consecutive HIV-positive clinic patients in Cameroon (250), United Kingdom (633), Ukraine (412), and Zimbabwe (245) donated venous blood (all countries) and finger prick blood (all except UK) for testing on SAMBA II. SAMBA II results were compared with simultaneous plasma results on the Abbott RealTime HIV-1 (Abbott Molecular, Des Plaines, IL) viral load assay and interpreted as either <1,000 RNA copies/ml or ≥1,000 RNA copies/ml. For 1,528 venous whole-blood samples tested on SAMBA II, overall percent agreement with the reference test at a cutoff HIV VL of ≥1,000 copies/ml was 96.9% (1,480/1,528; 95% confidence interval [CI], 95.9% to 97.7%), negative percent agreement was 97.7% (1,259/1,289; 95% CI, 96.7% to 98.4%), and positive percent agreement was 92.5% (221/239; 95% CI, 88.4% to 95.5%). For 854 finger prick samples, there was 95.0% (811/854; 95% CI, 93.3% to 96.3%) overall percent agreement, 98.0% (625/638; 95% CI, 96.5% to 98.9%) negative percent agreement, and 86.1% (186/216; 95% CI, 80.8% to 90.4%) positive percent agreement. These rose to 93.5% (82.1% to 98.6%), 97.6% (95.6% to 98.8%), and 95.6% (93.3% to 97.3%) after exclusion of aberrant results from the Ukraine center. These results show a high level of agreement between SAMBA-II and a laboratory-based assay. SAMBA-II has a performance that is suitable to use as a VL point-of-care assay in remote settings.

## Abstract (precis)

Remote areas of many low and middle income (LMI) countries have poor access to HIV viral load (HIV VL) testing. The SAMBA II (simple amplification-based assay) Semi-Q whole-blood test (Diagnostics for the Real World [DRW], Cambridge, UK) is a point-of-care assay, which uses leucodepletion technology to allow whole-blood testing in remote settings.

## INTRODUCTION

People living with HIV can live a near-normal length of life if they are treated with antiretroviral therapy (ART) and have a suppressed HIV viral load (VL). In 2014, the Joint United Nations Program on HIV and AIDS (UNAIDS) and partners set the 90-90-90 targets, aiming to diagnose 90% of all HIV-positive people, provide ART for 90% of those diagnosed, and achieve viral suppression for 90% of those treated by 2020 (1). To achieve the viral suppression target, there would need to be widespread availability of HIV VL testing. This is necessary to detect those patients with a raised HIV VL on ART who may have adherence problems or are developing ART resistance. Access to HIV VL testing for patients in low and middle income (LMI) countries has been problematic for a variety of reasons, including expense and lack of equipment, testing technology that is tied to sophisticated laboratory settings in cities and large towns, problems with maintenance of the testing machines, loss of access to reagents, the need for venous blood samples, and the logistics of testing patients who live large distances away from the laboratory facilities (2). As a result, in many LMI countries, only a minority of patients on ART receive monitoring of HIV VL. For instance, in a report from West and Central Africa, only 25% of people living with HIV were on ART and had an undetectable HIV VL, and even that was an estimate due to lack of accessible testing (3). When blood samples are taken for testing in rural areas, it can take 3 weeks or more to obtain the result with potential negative clinical consequences (4, 5). More recently, the testing of dried blood spot (DBS) samples has been attempted to overcome the problem of access to testing for patients living in hard-to-reach areas (6, 7). However, this approach still has the problems of delays due to transportation of samples to laboratories, test performance that is suboptimal when compared with that obtained from plasma samples, and the expense of using current testing technology (6–8).

In remote areas, the ideal HIV VL testing platform would be transportable, robust, produce a result while the patient was still in the clinic, low cost, and have a performance that is similar to laboratory-based assays (2, 5). Clinical decisions about ART use can then be made before the patient leaves the clinic, avoiding problems of loss to follow-up and the clinical and virological consequences of a delayed result and allowing immediate decisions to be made about adherence counselling and the need for ART change (9–12). While there has been some progress in point-of-care technology, current available platforms still require plasma samples and are often still tied to laboratory use (9–12). The SAMBA II HIV-1 Semi-Q whole-blood test is performed on a compact instrument about the size of a small microwave oven, which is robust and therefore easily transportable to remote areas. It works as a simple sample-in, result-out system using whole blood, thus avoiding the need for centrifugation to separate out plasma for testing, and it does not need to be placed in a sophisticated laboratory setting (12–14). It operates using whole-blood samples, including finger prick capillary blood, using internal microfilters, which remove white blood cells that otherwise cause false-positive results (13, 14). Results are available within 90 min, allowing clinical decisions to be made on the day of attendance. The result output is a binary <1,000 copies/ml or ≥1,000 copies/ml based on the WHO definition of potential ART treatment failure (≥1,000 copies/ml) (15).

The objectives of this study were to compare the performance of the SAMBA II Semi-Q whole-blood HIV viral load point-of-care assay against a plasma-based HIV VL assay using both venous blood and finger prick capillary blood in a range of settings in a developed country (United Kingdom), a middle-income country (Ukraine), and two developing countries (Cameroon and Zimbabwe). Testing at these sites would ensure that the assay would be used across a range of HIV subtypes, viral loads, and different environments. We hypothesized that the SAMBA II HIV-1 Semi-Q whole-blood test would perform well using whole blood compared with a standard plasma-based assay and therefore demonstrate its suitability for HIV VL testing.

## MATERIALS AND METHODS

### Study design.

This is a diagnostic accuracy study. The study took place in two phases. In phase 1, HIV VL was measured (<1,000 copies/ml or ≥1,000 copies/ml) on venous blood samples from consecutive people living with HIV (PLWH) attending an HIV service in London, UK, using the SAMBA II Semi-Q whole-blood HIV viral load point-of-care assay, and the results were compared with those obtained using an Abbott RealTime HIV-1 PCR assay. The performance of the SAMBA II HIV-1 Semi-Q whole-blood test was then optimized based on these results (results not given here). In phase 2, of which the results are reported here, HIV VL was measured on venous blood samples (London, Kiev, Dschang, and Harare) and finger prick capillary samples (Kiev, Dschang, and Harare) from consecutive PLWH attending these clinics. In the London part of the study, venous blood samples were rapidly transported to Diagnostics for the Real World (DRW) headquarters in Cambridge for testing. In the cases of Kiev, Dschang, and Harare, the SAMBA II HIV-1 Semi-Q whole-blood test was performed within the HIV clinic in conditions identical to those when used as a near-patient point-of-care assay.

### Participants. (i) Ethics approval and consent.

The study had prior approval from the following ethics committees: The NHS Integrated Research Application System, England; The Kyiv National Medical University, Ukraine; Harare city ethics committee and Harare hospital ethics board, Zimbabwe; and University of Dschang, Cameroon. All patients were given patient information leaflets prior to being enrolled, and written informed consent was obtained in their preferred language wherever possible.

### (ii) Eligibility criteria.

To be included in the study, patients had to be HIV-1 positive, >18 years of age, and able to read and understand forms and procedure. Patients were excluded from participation if they were younger than 18 years, pregnant, had active tuberculosis, or were unable to understand forms or procedure. All eligible adult HIV-positive patients attending the 4 participating HIV services consecutively for routine care in the enrollment period and who required a routine blood test were asked to donate two extra samples of venous blood in 3-ml EDTA bottles. In addition, at the Kiev, Cameroon, and Zimbabwe clinics, they were asked to agree to provide 100 μl (up to 6 drops) of capillary blood obtained through finger prick.

Patients were recruited in the following HIV clinics during these date ranges: Central Middlesex and Northwick Park Hospitals, London, England (30 October 2017 to 30 April 2018); Kiev City AIDS Center, Kiev, Ukraine (27 February 2018 to 15 November 2019); Hatcliffe Clinic and Harare Central Hospital, Harare, Zimbabwe (18 June 2018 to 4 October 2018); and University of Dschang, Cameroon (12 July 2018 to 21 September 2018).

### Test methods.

At the patient’s clinic visit, venous and capillary blood samples were obtained and tested on the SAMBA II Semi-Q whole-blood HIV viral load point-of-care assay as previously described (13, 14). This gives an HIV VL result as either <1,000 copies/ml or ≥1,000 copies/ml using an optical sensor reading of a test strip. In addition, routine viral load testing was performed on the corresponding plasma specimen for each patient according to their care requirements (Roche CAP/CTM v2 in Ukraine, Hologic Aptima HIV-1 Quant assay in United Kingdom and Cameroon, bioMérieux NucliSens EasyQ test in Zimbabwe). Two further 5-ml EDTA-anticoagulated venous blood samples were obtained for the purposes of this research and the extracted plasma stored for later testing with the Abbott RealTime PCR HIV VL assay to use as the comparator VL result with the SAMBA II HIV-1 Semi-Q whole-blood test VL results. The Abbott RealTime HIV-1 PCR assays were performed at accredited laboratories close to the research sites, excepting London samples, which were tested at Public Health England laboratories, Birmingham. The Abbott RealTime HIV-1 PCR assay was used as the comparator, as it works well across a range of HIV subtypes and has a low rate of overestimation of VL compared with that of other HIV VL assays (16). The HIV VL limit of 1,000 copies/ml to define “positivity” for the SAMBA II HIV-1 Semi-Q whole-blood test was chosen in keeping with the WHO definition of potential treatment failure with the patient requiring assessment for possible treatment change (15).

### Logistics of testing on SAMBA.

The SAMBA machines were present at each clinic site, and the VL testing was performed by technicians who had been trained by a certified trainer from DRW Ltd. A competency test was taken by each technician before they were allowed to use the assay “live.” The technicians were subsequently monitored by a local project coordinator with additional remote support by DRW scientists. The assay uses 100 μl of whole blood drawn into a standard-volume pipette from either an EDTA venous sample or a finger prick capillary sample. This sample is placed in the SAMBA machine, in which all processes, including microfiltration/leucodepletion, are fully automated leading to a result in 90 min. The result output is an optically scanned line on a test strip.

The mechanical and sample influences that potentially could go wrong were as follows: (i) failure to collect and load the capillary sample correctly, (ii) failure to load the venous sample correctly, (iii) incorrect maintenance of the SAMBA machine or use out of the temperature (2 to 37°C) and humidity (up to 80%) tolerance ranges, and (iv) incorrect storage of the reagent cartridges out of the temperature (2 to 37°C) and humidity (up to 80%) ranges.

### Analysis.

The limit of accuracy for the Abbott RealTime assay is ±0.3 log_10_ relative to the VL readout obtained (16), and ±0.3 log_10_ is the accepted diagnostic range of variability for PCR assays (17). For the purpose of this study, we therefore considered that any VL quantification by Abbott RealTime assay within 0.3 log_10_ of the SAMBA II HIV-1 Semi-Q whole-blood test cutoff of 1,000 (3 log_10_) copies/ml, corresponding to a range of 500 to 2,000 copies/ml, was concordant with the SAMBA II HIV-1 Semi-Q whole-blood test result.

### Statistical methods. (i) Sample size estimate.

At the time of planning this study, the WHO estimated that 15% of patients on ART exhibit treatment failure (18). This figure was used in estimating sample size. By enrolling 2,000 patients, approximately 300 samples with a VL of ≥1,000 copies/ml were expected (95% confidence interval [CI] width of 0.019 for the probability of concordance when the true value is 95%). This sample size also gives 90% power (two-sided 5% significance level) to rule out true concordance probabilities below 93% when the true value is 95%.

### (ii) Other statistics.

The differences between diagnostic agreement comparing venous and capillary samples tested on the SAMBA II HIV-1 Semi-Q whole-blood test and the Abbott RealTime HIV-1 PCR assay were assessed using Fisher’s exact test. The association between age, gender, and country and a VL of ≥1,000 copies/ml was performed using logistic regression. Initially, the association of each factor and each outcome was examined separately in a series of univariable analyses. Additionally, a multivariable analysis was performed where the association between the factors upon each outcome was examined jointly.

## RESULTS

### Participants.

All patients were HIV-positive and attending the clinics for HIV care, including assessment before starting and monitoring of antiretroviral therapy. The baseline demographics are given in Table 1.

**TABLE 1:** Summary of patient age and sex

Variable	No. from Cameroon (%) (*n* = 250)	No. from UK (%) (*n* = 633)^*a*^	No. from Ukraine (%) (*n* = 412)	No. from Zimbabwe (%) (*n* = 245)^*b*^	Total no. combined (*n* = 1,540)
Mean age (yr) ± SD	44.9 ± 10.7	47.6 ± 11.7	37.7 ± 9.4	40.1 ± 9.4	43.5 ± 10.1
Age category					
≤25	4 (1.6)	19 (3.0)	32 (7.7)	14 (5.7)	69 (4.5)
26–40	94 (37.6)	140 (22.1)	244 (59.1)	106 (43.2)	584 (38.1)
41–50	83 (33.2)	221 (34.9)	94 (22.8)	84 (34)	482 (31.5)
51–60	50 (20)	181 (28.6)	34 (8.2)	30 (12.2)	295 (19.3)
>60	19 (7.6)	72 (11.4)	8 (1.9)	3 (1.2)	102 (6.7)
Gender					
Female	175 (70)	298 (47.2)	142 (34)	140 (57.1)	745 (48.7)
Male	75 (30)	334 (52.8)	270 (66)	105 (42.9)	784 (51.3)

a Gender data missing for one patient.

b Age data missing for 8 patients.

The patients were given usual care and tested using the locally available HIV VL assay. The research results were not made available to the patients and did not influence clinical care. The prevalence of results ≥1,000 copies/ml are shown in Table 2. This reflects the availability of ART in each setting at the time of recruitment, with only 4% of test results ≥1,000 copies/ml in the United Kingdom and around 40% in the Ukraine.

**TABLE 2:** Prevalence of HIV VL results ≥1,000 copies/ml using the SAMBA II HIV-1 Semi-Q whole-blood test

Country	Venous blood		Capillary blood	
	No./total no.	% (95% CI)	No./total no.	% (95% CI)
Cameroon	29/249	11.7 (7.9–16.3)	25/250	10.0 (6.6–14.4)
UK	26/633	4.1 (2.7–6.0)		
Ukraine	161/402	40.1 (35.2–45.0)	144/398	36.2 (31.5–41.1)
Zimbabwe	35/244	14.3 (10.2–19.4)	28/206	13.6 (9.2–19.0)
All countries combined	251/1,528	16.4 (14.6–18.4)	197/854	23.1 (20.3–26.0)

### Enrollment and excluded results.

In London, 636 patients were enrolled, of which 3 (0.5%) samples were excluded from analysis due to technical failure. For Cameroon, out of 253 patients enrolled, 2 (0.8%) venous samples and 1 (0.4%) capillary sample were excluded for technical failure; 247 patients had paired venous and capillary samples tested, 3 had capillary samples tested only, and 2 had venous samples tested only. For Zimbabwe, 245 patients enrolled, 0 venous and 1 (0.4%) capillary sample was excluded for technical failure, 205 gave paired samples, 39 had venous samples tested only, and 1 patient had a capillary sample tested only. For Ukraine, 412 patients enrolled, 5 (1.2%) venous and 14 (3.4%) capillary results were excluded for technical failure, 390 paired sample results were obtained, 12 had venous sample results only, and 8 had capillary sample results only.

### Test results.

The performance of the assay at each test site is given in Table 3. There were few differences between the venous and capillary results. The only significant difference was for positive percent agreement in the Ukraine. This was found to be significantly higher for the venous blood samples than the capillary blood samples and was then reflected in the combined results for all countries. Further investigation of this discrepancy revealed that a technician at the Ukraine site had initially had difficulties with collection of the capillary samples, which was corrected midway through the research. This was not an issue at any of the other sites and was found to be a problem with one individual rather than an inherent design problem. We, therefore, did a further analysis excluding the Ukraine capillary results.

**TABLE 3:** Diagnostic agreement of the SAMBA II HIV-1 Semi-Q whole-blood test with the Abbott RealTime HIV-1 PCR assay in detecting VL ≥1,000 copies/ml

Country and statistic	Venous blood		Capillary blood		*P* value
	No./total no.	% (95% CI)	No./total no.	% (95% CI)	
Cameroon					
Positive % agreement	18/18	100 (81.5–100)	17/18	94.4 (72.7–99.9)	1.00
Negative % agreement	220/231	95.2 (91.6–97.6)	224/232	96.6 (93.3–98.5)	0.49
Total % agreement	238/249	95.6 (92.2–97.8)	241/250	96.4 (93.3–98.3)	0.66
UK					
Positive % agreement	18/22	81.8 (59.7–94.8)			
Negative % agreement	603/611	98.7 (97.4–99.4)			
Total % agreement	621/633	98.1 (96.7–99.0)			
Ukraine					
Positive % agreement	154/167	92.2 (87.1–95.8)	143/170	84.1 (77.7–89.3)	0.03
Negative % agreement	228/235	97.0 (94.0–98,8)	225/228	98.7 (96.2–99.7)	0.34
Total % agreement	382/402	95.0 (92.4–96.9)	368/398	92.5 (89.4–94.9)	0.20
Zimbabwe					
Positive % agreement	31/32	96.9 (83.8–99.9)	26/28	92.9 (76.5–99.1)	0.59
Negative % agreement	208/212	98.1 (95.2–99.5)	176/178	98.9 (96.0–99.9)	0.69
Total % agreement	239/244	98.0 (95.3–99.3)	202/206	98.1 (95.1–99.5)	1.00
All countries combined					
Positive % agreement	221/239	92.5 (88.4–95.5)	186/216	86.1 (80.8–90.4)	0.03
Negative % agreement	1,259/1,289	97.7 (96.7–98.4)	625/638	98.0 (96.5–98.9)	0.75
Total % agreement	1,480/1,528	96.9 (95.9–97.7)	811/854	95.0 (93.3–96.3)	0.03
All countries combined with Ukraine capillary results excluded					
Positive % agreement	221/239	92.5 (88.4–95.5)	43/46	93.5 (82.1–98.6)	1.00
Negative % agreement	1,259/1,289	97.7 (96.7–98.4)	400/410	97.6 (95.6–98.8)	0.85
Total % agreement	1,480/1,528	96.9 (95.9–97.7)	436/456	95.6 (93.3–97.3)	0.24

Analyses were performed to examine the association between patient demographics and the venous blood result (viral load ≥ 1,000 or not). A summary of both the univariable and multivariable analysis results are shown in Table 4.

**TABLE 4:** Associations with a viral load ≥ 1,000 on SAMBA II HIV-1 Semi-Q whole-blood test

Variable and category	Univariable			Multivariable	
	%	Odds ratio (95% CI)	*P* value	Odds ratio (95% CI)	*P* value
Country					
Cameroon	11.7	1	<0.001	1	<0.001
UK	4.1	0.32 (0.19–0.56)		0.32 (0.18–0.57)	
Ukraine	40.1	5.07 (3.28–7.83)		4.54 (2.86–7.19)	
Zimbabwe	14.3	1.27 (0.75–2.15)		1.15 (0.67–1.98)	
Age (yr)					
≤25	33.3	1	<0.001	1	0.06
26–40	23.0	0.60 (0.35–1.03)		0.53 (0.29–0.98)	
41–50	12.1	0.27 (0.15–0.49)		0.39 (0.20–0.75)	
51–60	9.6	0.21 (0.11–0.40)		0.44 (0.21–0.90)	
>60	7.8	0.17 (0.07–0.41)		0.42 (0.16–1.10)	
Gender					
Female	14.2	1	0.02	1	0.60
Male	18.7	1.39 (1.06–1.83)		1.09 (0.80–1.49)	

The analysis results suggested strong evidence of a difference in test results between the four countries, in both the univariable and multivariable results. Test results were over 1,000 in 40% of subjects from the Ukraine but in only 4% from the United Kingdom, although this merely reflects the relative availability of ART at the centers.

There was a highly significant difference in test result between age groups in the univariable analysis. The occurrence of a test result above 1,000 decreased with increasing age. After adjusting for the other variables, particularly country, the difference between age categories was less, with the differences being only of borderline statistical significance (*P* = 0.06). The ≤25 age band still had the highest occurrence of a high test result, but the difference between older age categories was lower after accounting for other variables.

There was a significant difference between genders in the univariable analysis. However, this difference was not at all significant in the multivariable analysis. Thus, the initial significant finding is likely to be due to country differences between males and females. The supplemental material gives further results of the diagnostic performance of the SAMBA II assay in detecting VLs of >1,000 copies/ml for each country separately and combined using a tiebreaker discrepancy analysis. There is also a table of associations with a concordant test result.

## DISCUSSION

This study shows that when tested in a range of geographical settings, the SAMBA II HIV-1 Semi-Q whole-blood test has a level of performance in detecting potential ART treatment failure at the WHO-defined level of ≥1,000 copies/ml (15) that makes it suitable for use in remote settings. This applies to both capillary and venous blood samples.

Currently, there is no suitable/accurate technology for use as a near-patient point-of-care assay for HIV VL in very remote settings. In low-income countries, patients would have to be in cities or large towns to be near a testing laboratory to get accurate results (2, 11, 19). Even then, phlebotomy for venous blood is required, followed by centrifugation for plasma separation (2, 11, 19, 20) adding delays and complexity to the process. For patients living in remote places, the logistics of taking blood samples, getting a plasma sample to the regional laboratory, and obtaining the results are great, and delays of 3 weeks are not unusual with many patients not having access to testing (2–5). Dried blood spot (DBS) samples have been used for testing patients in remote places, but these still suffer the disadvantage of having to be transported to regional laboratory facilities with inherent delays (6–8). DBS performance in testing HIV VL is also variable, as the volume of blood on the card is not fixed, with some studies showing relatively poor performance (6–8). There are other whole-blood HIV viral load point-of-care assays available, but they suffer from poor performance with low specificity (<80%) due to the contamination of the specimen with nucleated blood cells and high error rates (12, 21). Similarly, plasma-based viral load point-of-care assays also have a specificity of <80% (22). The SAMBA II HIV-1 Semi-Q whole-blood test overcomes these disadvantages by removing white cells using microfilters. Our study shows good levels of overall percent agreement (>95%), positive percent agreement (>86%), and negative percent agreement (>97%) when compared with those of a plasma-based, laboratory-based standard assay, meaning that clinically relevant results are obtained even when using finger prick capillary blood. Although overall positive percent agreement of the finger prick capillary blood was 86.1%, this rose to >92.5% when excluding the Ukraine capillary results where operator error was identified. There is an interassay variability in quantification results of about 5% for all HIV viral load assays, especially at higher viral loads (23–25). Therefore, the differences between the SAMBA II HIV-1 Semi-Q whole-blood test and the standard comparator assay can be partly explained by normal interassay variation.

### Study limitations.

Due to logistics of site setup, enrollment took place at the four sites at different times over an 18-month period, although the results at each site remain blinded and each site was unaware of the results at other sites. There could be some differences in the sites technical use of the SAMBA machine, although it is a straightforward sample-in, result-out technology. The only significant difference between venous and capillary results was the reduced positive percentage agreement of capillary samples compared with that of venous samples at the Ukraine site. This was found to be due to operator error of one individual collecting the capillary samples. A difference in positive percentage agreement was seen when all site results were combined but was due to the influence of the Ukraine results. This disappeared when Ukraine results were excluded. Negative percentage agreement was greater for the capillary blood results when all sites were combined but not at all individual sites, although the difference is numerically small. There was a trend toward younger age and VL of ≥1,000 copies/ml, but this probably just reflects enrollment characteristics rather than biological differences in test performance. Testing of HIV VL on SAMBA and the Abbott assay was separated in time and location so that there could be no linkage until the time of collation and analysis of results, which reduced the risk of potential bias in recording the SAMBA results.

The results are generalizable in that we used four different clinic settings to perform the research. The most prevalent HIV subtypes are B (plus C, A, and D) in the UK, A (plus B) in Ukraine, CRF02_AG in Cameroon, and C in Zimbabwe (26–29). This meant that the assay was performed on a range of HIV subtypes. The study was also undertaken in settings identical to those where it is intended to be used, i.e., in HIV clinics in LMI countries.

The simple design of the SAMBA technology allows sample-in and results-out in about 90 min and is already in use in remote settings in low- and middle-income countries (30). As the SAMBA II HIV-1 Semi-Q whole-blood test works on whole blood, there is no need for phlebotomy, centrifugation, and plasma separation. It therefore is a true point-of-care assay with clinicians being able to access the results before the patients leave the clinic.

### Conclusion.

The SAMBA II HIV-1 Semi-Q whole-blood test has a performance that is comparable to laboratory-based assays.

## Supplementary Material

Supplemental file 1
