# Supplementary material for: Study To Evaluate the Performance of a Point-of-Care Whole-Blood HIV Viral Load Test (SAMBA II HIV-1 Semi-Q Whole Blood)
Source: J Clin Microbiol. 2021 Feb 18;59(3):e02555-20. doi: 10.1128/JCM.02555-20 (PMC8106730; doi:10.1128/JCM.02555-20)
Supplement: Supplemental file 1 [file JCM.02555-20-s0001.pdf]

Supplementary files

In the original protocol, there was provision for a tie-breaker viral load measurement with a third assay. In cases of discrepant (non-agreement of) results between the SAMBA and Abbot assays, a further viral load assay was performed using an alternative commercial assay ('tie-breaker'), as available locally: either the Holigic Aptima or Roche Taqman assays. The SAMBA result for analysis was then taken as 'discrepant' if the tie breaker agreed with the Abbott result or 'concordant' if the tie breaker agreed with the SAMBA result. If a tie-breaker test was not done after an initial discrepant result then the result was excluded from the analysis. The per-protocol analysis using this tie-breaker data is given in Table S1.

Table S1: Diagnostic performance of the SAMBA II assay in detecting VL >1000cps/ml for each country separately using tie-breaker discrepancy analysis.

Country /	Venous blood		Capillary blood		P (*)
statistic	n / N	% (95% CI)	n / N	% (95% CI)	
**<u>Cameroon</u>**					
Sensitivity	19 / 19	100.0 (82.4, 100.0)	18 / 19	94.7 (74.0, 99.9)	1.00
Specificity	220 / 230	95.7 (92.1, 97.9)	224 / 231	97.0 (93.9, 98.8)	0.58
PPV	19 / 29	65.5 (45.7, 82.1)	18 / 25	72.0 (50.6, 87.9)	-
NPV	220 / 220	100.0 (98.3, 100.0)	224 / 225	99.6 (97.5, 100.0)	-
Concordance	239 / 249	96.0 (92.7, 98.1)	242 / 250	96.8 (93.8, 98.6)	0.79
**<u>UK</u>**					
Sensitivity	21 / 24	87.5 (67.6, 97.3)	-	-	-
Specificity	604 / 609	99.2 (98.1, 99.7)	-	-	-
PPV	21 / 26	80.8 (60.6, 93.4)	-	-	-
NPV	604 / 607	99.5 (98.6, 99.9)	-	-	-
Concordance	625 / 633	98.7 (97.5, 99.5)	-	-	-
**<u>Ukraine</u>**					
Sensitivity	156 / 166	93.4 (89.2, 97.1)	144 / 167	86.2 (80.0, 91.1)	**0.02**
Specificity	231 / 236	97.9 (95.1, 99.3)	229 / 231	99.1 (96.9, 99.9)	0.45
PPV	156 / 161	96.9 (92.9, 99.0)	144 / 146	98.6 (95.1, 99.8)	-
NPV	231 / 241	95.8 (92.5, 98.0)	229 / 252	90.9 (86.6, 94.1)	-
Concordance	387 / 402	96.3 (93.9, 97.9)	373 / 398	93.7 (90.9, 95.9)	0.09

**Zimbabwe**					
Sensitivity	31 / 31	100.0 (88.8, 100.0)	26 / 26	100 (86.8, 100.0)	1.00
Specificity	209 / 213	98.1 (95.3, 99.5)	178 / 180	98.9 (96.0, 99.9)	1.00
PPV	31 / 35	88.6 (73.3, 96.8)	26 / 28	92.3 (76.5, 99.1)	-
NPV	209 / 209	100.0 (98.3, 100.0)	178 / 178	100 (97.9, 100.0)	-
Concordance	240 / 244	98.4 (95.9, 99.6)	204 / 206	99.0 (96.5, 99.9)	1.00
**All countries**					
Sensitivity	227 / 240	94.6 (90.9, 97.1)	188 / 212	88.7 (83.6, 92.6)	-
Specificity	1264 / 1288	98.1 (97.2, 98.8)	631 / 642	98.3 (97.0, 99.1)	-
PPV	227 / 251	90.4 (86.1, 93.7)	188 / 199	94.5 (90.3, 97.2)	-
NPV	1264 / 1277	99.0 (98.3, 99.5)	631 / 655	96.3 (94.6, 97.6)	-
Concordance	1491 / 1528	97.6 (96.7, 98.3)	819 / 854	95.9 (94.3, 97.1)	-

Additional analyses considered the association between patient demographics and test outcomes. Analyses were performed to examine factors associated with concordance between the assay results and the per-protocol tie-breaker result. The analyses were based on the venous blood results. Table S2 shows a summary of both the univariable and multivariable results. For the categorical variables, the first figures show the percentage concordance within each category. Subsequently, for each analysis, the size of effect of each variable is reported as an odds ratio, along with corresponding confidence interval. These show the odds of a concordant result in each category relative to the odds in a baseline category.

Both the univariable and multivariable analyses suggested no strong evidence that either age or sex was associated with the concordance of the results between venous samples and the gold standard. There was slight evidence of a difference between age categories in the univariable analyses, but this did not remain after adjusting for the other factors in the multivariable analysis. The univariable analysis suggested a significant difference in concordance between the four countries. Concordance was highest in the UK and Zimbabwe, with lower values in Cameroon and the Ukraine. The odds of a concordant result were 3 times higher in the UK than in Cameroon. After adjusting for the age and sex of the participants in the multivariable analysis, the difference between countries did not quite reach statistical significance (p=0.1). The trend was still for higher concordance levels in the UK and Zimbabwe compared to Cameroon and the Ukraine.

Table S2: Associations with a concordant test result

Variable	Category	Univariable			Multivariable	
		%	OR (95% CI)	P-value	OR (95% CI)	P-value
Country	Cameroon	96.0%	1	**0.03**	1	0.10
	UK	98.7%	3.27 (1.27, 8.38)		2.78 (1.06, 7.33)	
	Ukraine	96.3%	1.08 (0.48, 2.44)		1.12 (0.46, 2.73)	
	Zimbabwe	98.4%	2.51 (0.78, 8.12)		2.42 (0.74, 7.97)	
Age	≤ 25	97.0%	1	0.08	1	0.25
	26-40	96.2%	0.79 (0.18, 3.44)		0.89 (0.20, 3.92)	
	41-50	98.8%	2.47 (0.49, 12.5)		2.33 (0.45, 12.0)	
	51-60	98.6%	2.26 (0.40, 12.6)		1.95 (0.34, 11.3)	
	> 60	97.1%	1.03 (0.17, 6.34)		0.87 (0.13, 5.63)	
Gender	Female	97.2%	1	0.35	1	0.42
	Male	97.9%	1.37 (0.71, 2.65)		1.33 (0.66, 2.69)	
